# Causal relationship between gut microbiota and diabetic complications: a two-sample Mendelian randomization study

**DOI:** 10.1186/s13098-024-01424-7

**Published:** 2024-08-20

**Authors:** Jinya Liu, Yuanyuan Chen, Cheng Peng

**Affiliations:** 1 Department of Burn and Plastic Surgery, The Third Xiangya Hospital of Central South University, Changsha, 410013 Hunan China; 2 Department of Cardiology, The Third Xiangya Hospital of Central South University, Changsha, 410013 Hunan China

## Abstract

**Background:**

Imbalances in gut microbiota (GM) have been proposed as a potential contributing factor to diabetic complications; however, the causal relationship remains incompletely understood.

**Methods:**

Summary statistics were obtained from genome-wide association studies (GWAS) of 196 gut microbial taxa, including 9 phyla, 16 classes, 20 orders, 32 families, and 119 genera. These data were then analyzed using mediation Mendelian randomization (MR) analyses to explore the potential mediating effect of diabetes complications risk factors on the relationship between gut microbiota and specific diabetic complications such as diabetic kidney disease (DKD), ketoacidosis, and diabetic retinopathy (DR).

**Results:**

In our Mendelian analysis, we observed negative associations between Bifidobacterial order and Actinomycete phylum with DKD in type 1 diabetes (T1D) as well as early DKD in T1D. Conversely, these taxa showed positive associations with ketoacidosis in type 2 diabetes (T2D). In reverse Mendelian analysis, we found that DR in both T1D and T2D as well as ketoacidosis in T2D affected the abundance of Eubacterium fissicaten genus and LachnospiraceaeUCG010 family within the gut microbiota.

**Conclusions:**

Our findings provide compelling evidence for causal relationships between specific GM taxa and various diabetes complications. These insights contribute valuable knowledge for developing treatments targeting diabetes-related complications.

**Supplementary Information:**

The online version contains supplementary material available at 10.1186/s13098-024-01424-7.

## Introduction

Diabetes is a prevalent global epidemic. The number of diabetic patients is increasing significantly, estimated to reach 700 million by 2045 [1]. In addition to the high blood glucose level and insulin resistance, many tissues are involved dysfunctions, for example, devastating microvascular complications (diabetic retinopathy, neuropathy, and renal disease) and significant macrovascular consequences such as cardiovascular disease [2]. Besides, diabetic patients are experiencing various complications.Current research reveals that the complex mechanism of diabetes complications, including epigenetics, immunity and neurodegeneration [3–5]. Therefore, researchers proposed various preventive measures and treatments [6]. Curiously, diabetic patients develop different diabetic complications, and understanding the mechanism remains a mystery. Thus, whether there exist novel factors contributing to differences in individuals remains unknown.

The gut microbiota (GM) refers to the microbial communities residing in the intestine, which live in symbiosis with the host and consist of fungi, viruses, and bacteria. Notably, bacteria constitute 98% of the gut microbiota [7]. Animal studies on the gut microbiota have revealed a causal role in the development of type 2 diabetes (T2D), obesity, and insulin resistance [8]. However, there remains a gap in understanding the relationship between the gut microbiota and diabetic complications.

Mendelian randomization (MR) methods employ single nucleotide polymorphisms (SNPs) as instrumental variables (IVs) to model and infer causal effects, mitigating the confounding variables that could potentially compromise the causal inference of exposure and outcome in previous epidemiological or observational studies. By leveraging the random assignment of SNPs during conception, the Mendelian randomization strategy effectively eliminates confounding circumstances [7]. Moreover, since heredity is irreversible, reverse causation interference can be ruled out. Several researchers have identified an association between gut microbiota and diabetic retinopathy [9]. Leveraging the high reliability of MR in establishing causality, we aim to uncover a causal relationship between diabetic complications and intestinal flora to offer novel perspectives on prevention and treatment.

## Methods

### Data resources

#### Exposure data

Genetic variances related to gut microbiota composition were selected from the MiBioGen consortium GWAS data, which were genome-wide genotypes and 16S fecal microbiome data from 18,340 individuals. This study was a large-scale, multi-ethnic, genome-wide meta-analysis of gut microbiota from 24 cohorts from the USA, Canada, Israel, South Korea, Germany, Denmark, the Netherlands, Belgium, Sweden, Finland and the UK. 211 taxa composed of 131 genera, 35 families, 20 orders, 16 classes, and 9 phyla.

#### Outcome data

##### Diabetic kidney disease

The summary data of diabetic kidney disease in patients with T1D were obtained from JDRF Diabetic Nephropathy Collaborative Research Initiative GWAS dataset including up to 19,300 participants [10]. Early DKD in T1D referred to patients with minimum T1D duration more than 5 years with microalbuminuria (AER ≥ 20–< 200 μg/min OR ≥ 30–< 300 mg/24 h OR an ACR of ≥ 30–< 300 mg/g OR ≥ 3.4–≤ 34 mg/mmol). Late DKD in T2D meant patients with macroalbuminuria and ESKD (end-stage kidney disease) (AER ≥ 200 μg/min OR ≥ 300 mg/24 h OR ACR ≥ 300 mg/g OR > 34 mg/mmol OR positive for albuminuria by dipstick ≥ 1 + OR eGFR < 15 ml/min per 1.73m2 OR dialysis OR renal transplant) with minimum T1D duration of 10 years. DKD in T1D included individuals with early or late diabetic kidney disease in T1D. Data of individuals with diabetic kidney disease of T2D were acquired form the Scania Diabetes Registry (SDR) [11], Genetics of Diabetes Audit and Research in Tayside Scotland (GoDARTS) study [12], Steno Diabetes Centre [13], and Bergamo Nephrologic Diabetes Complications Trial (BENEDICT) A and B studies [14], and GWAS study was by van Zuydam et al. [15]. based on 5717 individuals. Early DKD in T2D is patients in T2D tested with Microalbuminuria (AER ≥ 20 AND < 200 μg/min OR AER ≥ 30 AND < 300 mg/24 h OR ACR ≥ 2.5/3.5 AND < 25/35 mg/mmol for men/women). Late DKD in T2D was patients both with macroalbuminuria and ESKD among individual with T2D (AER ≥ 200 μg/min OR AER ≥ 300 mg/24 h OR ACR ≥ 25/35 mg/mmol for men/women OR eGFR < 15 ml/min per 1.73 m^2^ OR undergoing dialysis OR having a renal transplant). DKD in T2D included individuals with early and late diabetic kidney disease in T2D.

##### Diabetic ketoacidosis

The GWAS level data resource of diabetic ketoacidosis was extracted from UK Biobank dataset of 456,348 samples [16]. Diabetic ketoacidosis was selected according to the International Classification of Diseases, tenth revision (ketoacidosis in T1D was PheCode 250.11) and ketoacidosis in T2D was PheCode 250.21).

##### Diabetic retinopathy

There were 456,348 patients with diabetes mellites of the GWAS summary data extracted from the UK Biobank samples [16]. Diabetes mellites was also selected according to the International Classification of Diseases, tenth revision (T1D was PheCode 250.13) and T2D is PheCode 250.23). T1D and T2D diabetic retinopathy were defined as type I and type II IDDM (juvenile type), not stated as uncontrolled, with ophthalmic manifestations, respectively.

### Statistical analysis

#### Instrumental variables (IVs) selection

**Fig. 1:**
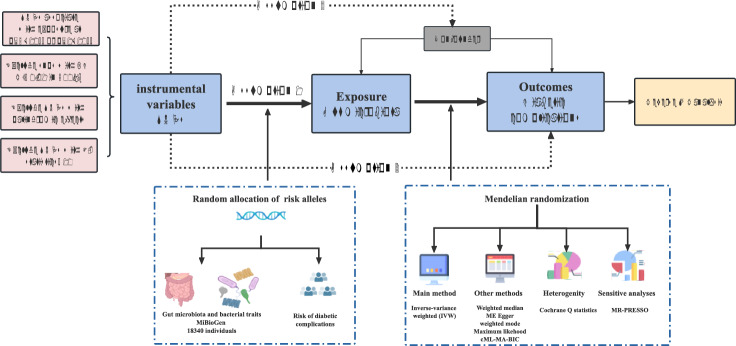
Design of the study. According to strict selection criteria, we obtained genetic correlation data from the summarized GWAS data for exposure and outcome. *GWAS* genome wide association study, *SNPs* single nucleotide polymorphisms, *MR* Mendelian randomization

The flowchart of this mendelian randomization study is show in Fig. 1. A total of 211 bacterial taxa were tasted according to phylum, class, order, family, and genus at five level. We deleted unknown 5 gut microbiotas and left 196 (9 phyla, 16 classes, 20 orders, 32 families, 119 genus). We used the following selection criteria were used to choose the instrumental variables: (1) two thresholds were used to select the potential IVs. A set of IVs was chosen based on the locus-wide significance threshold p-value < 5 × 10^–8^, the other was selected according to the locus-wide significance threshold p-value < 1 × 10^–6^; (2) 1000 Genomes project European samples data were used as the reference panel to calculate the linkage disequilibrium (LD) between the SNPs, and among those SNPs that had R^2^ < 0.01 (clumping window size = 500 kb), only the SNPs with the lowest P-values were retained; (3) SNPs with minor allele frequency (MAF) ≤ 0.01 were removed; (4) when palindromic SNPs existed, the forward strand alleles were inferred; and (5) the F statistic value of SNPs was no more than 10 (weakness instrumental effect) were removed potential IVs.

#### MR analyses and sensitivity analyses

We performed MR analyses to study the causal relations between the gut microbiota composition and common diabetic complications. In the primary stage, we used inverse-variance weighted (IVW) as the main study method in this study. This method could obtain an unbiased causal estimate if there was no horizontal pleiotropy and heterogeneity [17]. Notably, in case that heterogeneity and horizontal pleiotropy absented in the MR study, the estimate result of ML was consistent to IVW. In addition, four other complementary MR approaches were performed to invalidate the causal relationships, including weighted median, MR Egger, weighted mode and cML-MA-BIC. The MR-PRESSO analysis was used to evaluate the horizontal pleiotropy. The MR-Egger regression and MR-PRESSO analysis are based on the assumption that the instrument strength is independent of the direct effect (InSIDE). The MR-Egger regression was able to estimate the horizontal pleiotropy through the intercept. There was no horizontal pleiotropy and the result was similar to IVW in the MR study, when the intercept term was zero [18]. The MR-PRESSO analysis detects and attempts to reduce horizontal pleiotropy by removing significant outliers, depending on InSIDE assumptions [19]. Thus, we introduce a constrained maximum likelihood and model averaging-based MR method to control correlated and uncorrelated pleiotropic effects, namely, cML-MA-BIC, which do not depend on InSIDE assumptions [20].

We used Cochran’s Q statistics in IVW methods to determine the heterogeneity of IVs. Q statistics significant at a p-value < 0.05 can imply the presence of heterogeneity.

To further confirm the causal effects of diabetic complications on gut microbiota features, we performed reverse MR analyses, in which diabetic complications were set as exposure factor and gut microbiota were outcome factor.

#### Multiple testing correction

After MR analyses, we perform Multiple testing correction threshold at each feature level (phylum, class, order, family, and genus), defined as p < 0.05/n (where n is the effective number of independent bacterial taxa at the corresponding taxonomic level) in the final stage.

## Results

### Primary MR results

**Fig. 2:**
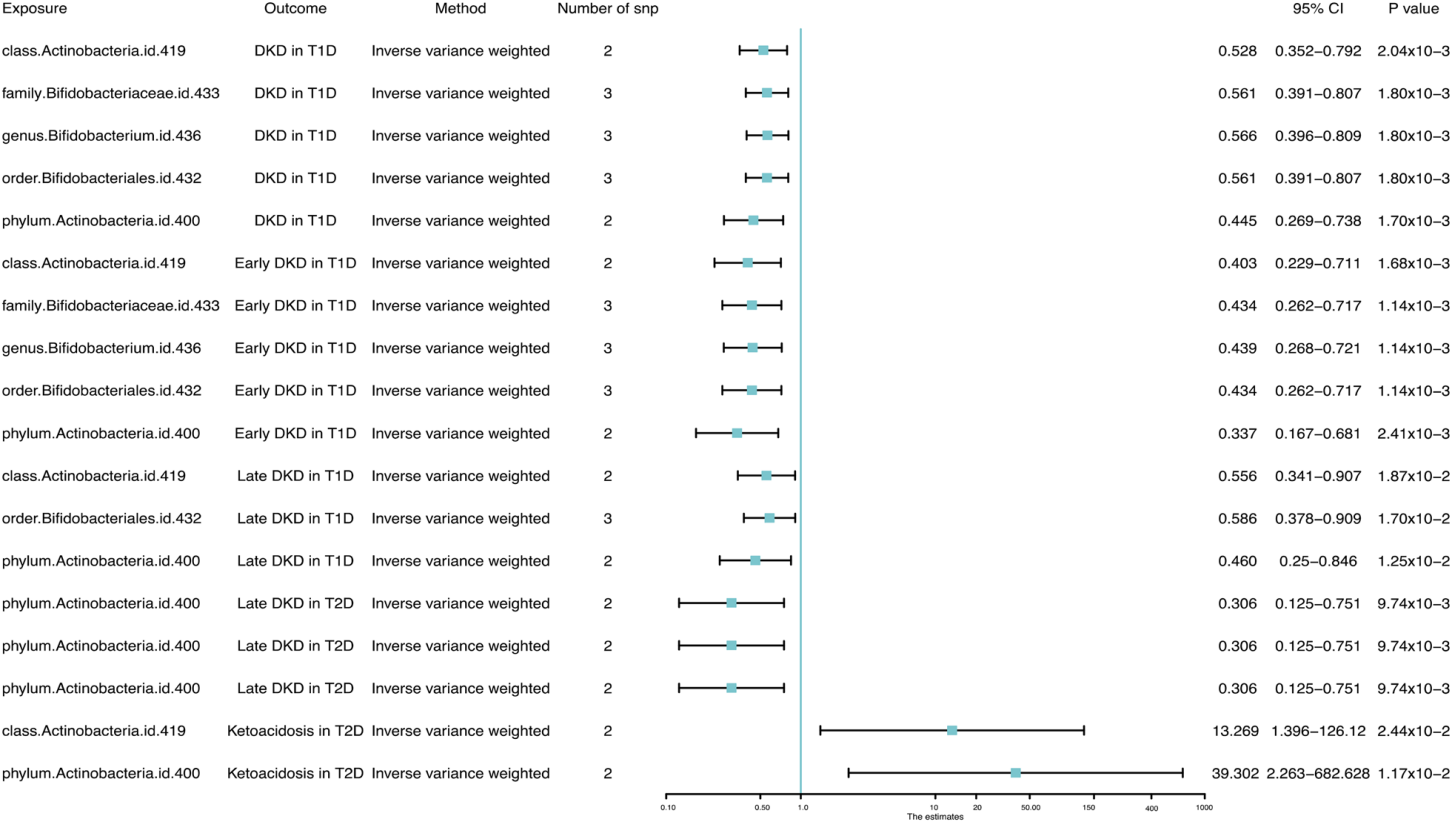
Forest plot of causal effects between gut microbiota and diabetic complication risk (p < 5 × 10^–8^). Forrest plot representing the MR estimats and 95% CI values of the causal effects of gut microbiota (as exposure) and diabetic complications (as outcomes), instrumental variables selected by p-value < 5 × 10^–8^, as estimated using Inverse-variance weighted. 95% CI 95% confidence interval, SNP single nucleotide polymorphism

**Fig. 3:**
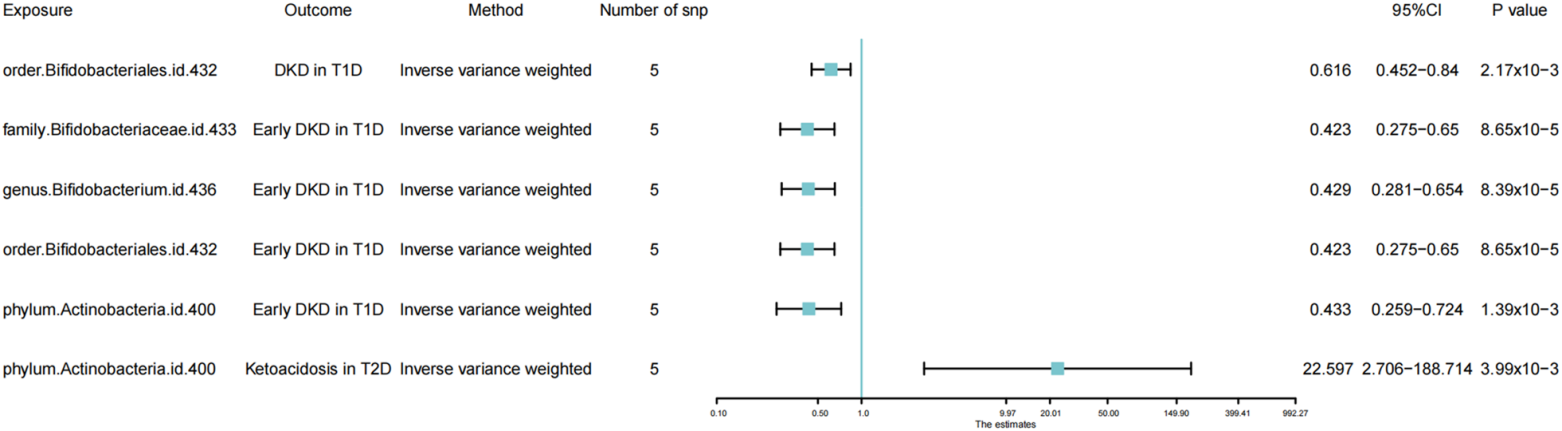
Forest plot of causal effects between gut microbiota and diabetic complication risk (p < 1 × 10^–6^). Forrest plot representing the MR estimats and 95% CI values of the causal effects of gut microbiota (as exposure) and diabetic complications (as outcomes), instrumental variables selected by p-value < 1 × 10^–6^, as estimated using Inverse-variance weighted. 95% CI 95% confidence interval, SNP single nucleotide polymorphism

Six methods and two thresholds were used in our research. According to the threshold of (p < 5 × 10^–8^), we obtained 5 bacterial (Table 1). Besides, we selected the IVW analysis methods and presented them using Forest plots (Fig. 2). Phylum.Actinobacteria (IVW: OR = 0.445, 95% CI 0.269–0.738, p = 0.0017) and class.Actinobacteria (IVW: OR = 0.528, 95% CI 0.269–0.738, p = 0.00204) reduced the risk incidence of DKD in T1D (including early and late DKD), and phylum.Actinobacteria (IVW: OR = 0.306, 95% CI 0.125–0.751, p = 0.00974) had a protective effect on late DKD in T2D, as well. However, the MR results indicated that phylum.Actinobacteria (IVW: OR = 13.269, 95% CI 1.396–126.12, p = 0.02442) and class.Actinobacteria (IVW: OR = 39.302, 95% CI 2.263–682.628, p = 0.01171) increased the risk of development on DKA in T2D. Besides, family.Bifidobacteriaceae (IVW: OR = 0.561, 95% CI 0.391–0.807, p = 0.0018), genus.Bifidobacterium (IVW: OR = 0.566, 95% CI 0.396–0.809, p = 0.0018) and order.Bifidobacteriales IVW: OR = 0.561, 95% CI 0.391–0.807, p = 0.0018) showed a protective effect on DKD in T1D, especially in early DKD in T1D. Next, we adjusted the threshold (p < 1 × 10^–6^) and performed the same steps as in the above analyses (Table 2 and Fig. 3), obtaining that family.Bifidobacteriaceae (IVW: OR = 0.423, 95% CI 0.275–0.65, p = 8.65 × 10^–5^), genus.Bifidobacterium (IVW: OR = 0.429, 95% CI 0.281–0.654, p = 8.39 × 10^–5^) and order.Bifidobacteriales (IVW: OR = 0423, 95% CI 0.275–0.65, p = 8.65 × 10^–5^) had a protective effect against early DKD in T1D, and that phylum.Actinobacteria (IVW: OR = 22.597, 95% CI 2.706–188.714, p = 0.003987) continued to increase the risk of DKA in T2D.

**Table 1:** Mendelian randomisation (MR) results of causal effects between gut microbiota and diabetic complication risk (p < 5 × 10^–8^)

Gut microbiota (exposure)	Diabetic complications (outcome)	Methods	Number of SNPs	Beta	SE	p-value	OR	95% CI
class.Actinobacteria.id.419	DKD in T1D	IVW	2	− 0.6391	0.20725	0.00204	0.528	0.352–0.792
class.Actinobacteria.id.419	DKD in T1D	Maximum likelihood	2	− 0.6392	0.21308	0.0027	0.528	0.348–0.801
class.Actinobacteria.id.419	DKD in T1D	cML-MA-BIC	2	− 0.6392	0.21308	0.0027	0.528	0.348–0.801
family.Bifidobacteriaceae.id.433	DKD in T1D	IVW	3	− 0.5774	0.18502	0.0018	0.561	0.391–0.807
family.Bifidobacteriaceae.id.433	DKD in T1D	Weighted median	3	− 0.6049	0.21494	0.00489	0.546	0.358–0.832
family.Bifidobacteriaceae.id.433	DKD in T1D	Maximum likelihood	3	− 0.5774	0.18938	0.0023	0.561	0.387–0.814
family.Bifidobacteriaceae.id.433	DKD in T1D	cML-MA-BIC	3	− 0.5777	0.18949	0.0023	0.561	0.387–0.814
genus.Bifidobacterium.id.436	DKD in T1D	IVW	3	− 0.569	0.18228	0.0018	0.566	0.396–0.809
genus.Bifidobacterium.id.436	DKD in T1D	Maximum likelihood	3	− 0.569	0.18648	0.00228	0.566	0.393–0.816
genus.Bifidobacterium.id.436	DKD in T1D	cML-MA-BIC	3	− 0.5692	0.18659	0.00228	0.566	0.393–0.816
order.Bifidobacteriales.id.432	DKD in T1D	IVW	3	− 0.5774	0.18502	0.0018	0.561	0.391–0.807
order.Bifidobacteriales.id.432	DKD in T1D	Weighted median	3	− 0.6049	0.21494	0.00489	0.546	0.358–0.832
order.Bifidobacteriales.id.432	DKD in T1D	Maximum likelihood	3	− 0.5774	0.18938	0.0023	0.561	0.387–0.814
order.Bifidobacteriales.id.432	DKD in T1D	cML-MA-BIC	3	− 0.5777	0.18949	0.0023	0.561	0.387–0.814
phylum.Actinobacteria.id.400	DKD in T1D	IVW	2	− 0.8087	0.25767	0.0017	0.445	0.269–0.738
phylum.Actinobacteria.id.400	DKD in T1D	Maximum likelihood	2	− 0.809	0.2688	0.00261	0.445	0.263–0.754
phylum.Actinobacteria.id.400	DKD in T1D	cML-MA-BIC	2	− 0.809	0.2688	0.00261	0.445	0.263–0.754
class.Actinobacteria.id.419	Early DKD in T1D	IVW	2	− 0.9078	0.28889	0.00168	0.403	0.229–0.711
class.Actinobacteria.id.419	Early DKD in T1D	Maximum likelihood	2	− 0.9078	0.29732	0.00226	0.403	0.225–0.722
class.Actinobacteria.id.419	Early DKD in T1D	cML-MA-BIC	2	− 0.9078	0.29732	0.00226	0.403	0.225–0.722
family.Bifidobacteriaceae.id.433	Early DKD in T1D	IVW	3	− 0.8349	0.25653	0.00114	0.434	0.262–0.717
family.Bifidobacteriaceae.id.433	Early DKD in T1D	Weighted median	3	− 0.8624	0.29812	0.00382	0.422	0.235–0.757
family.Bifidobacteriaceae.id.433	Early DKD in T1D	Maximum likelihood	3	− 0.8347	0.2632	0.00152	0.434	0.259–0.727
family.Bifidobacteriaceae.id.433	Early DKD in T1D	cML-MA-BIC	3	− 0.8349	0.26333	0.00152	0.434	0.259–0.727
genus.Bifidobacterium.id.436	Early DKD in T1D	IVW	3	− 0.8223	0.25272	0.00114	0.439	0.268–0.721
genus.Bifidobacterium.id.436	Early DKD in T1D	Maximum likelihood	3	− 0.8221	0.25914	0.00151	0.439	0.264–0.730
genus.Bifidobacterium.id.436	Early DKD in T1D	cML-MA-BIC	3	− 0.8224	0.25928	0.00151	0.439	0.264–0.730
order.Bifidobacteriales.id.432	Early DKD in T1D	IVW	3	− 0.8349	0.25653	0.00114	0.434	0.262–0.717
order.Bifidobacteriales.id.432	Early DKD in T1D	Weighted median	3	− 0.8624	0.29812	0.00382	0.422	0.235–0.757
order.Bifidobacteriales.id.432	Early DKD in T1D	Maximum likelihood	3	− 0.8347	0.2632	0.00152	0.434	0.259–0.727
order.Bifidobacteriales.id.432	Early DKD in T1D	cML-MA-BIC	3	− 0.8349	0.26333	0.00152	0.434	0.259–0.727
phylum.Actinobacteria.id.400	Early DKD in T1D	IVW	2	− 1.0867	0.35812	0.00241	0.337	0.167–0.681
phylum.Actinobacteria.id.400	Early DKD in T1D	Maximum likelihood	2	− 1.087	0.37255	0.00353	0.337	0.162–0.700
phylum.Actinobacteria.id.400	Early DKD in T1D	cML-MA-BIC	2	− 1.087	0.37255	0.00353	0.337	0.162–0.700
class.Actinobacteria.id.419	Late DKD in T1D	IVW	2	− 0.5873	0.24984	0.01874	0.556	0.341–0.907
class.Actinobacteria.id.419	Late DKD in T1D	Maximum likelihood	2	− 0.5873	0.25395	0.02073	0.556	0.338–0.914
class.Actinobacteria.id.419	Late DKD in T1D	cML-MA-BIC	2	− 0.5873	0.25395	0.02073	0.556	0.338–0.914
order.Bifidobacteriales.id.432	Late DKD in T1D	IVW	3	− 0.5337	0.22363	0.017	0.586	0.378–0.909
order.Bifidobacteriales.id.432	Late DKD in T1D	Maximum likelihood	3	− 0.5337	0.22677	0.0186	0.586	0.376–0.915
order.Bifidobacteriales.id.432	Late DKD in T1D	cML-MA-BIC	3	− 0.5339	0.22687	0.01861	0.586	0.376–0.915
phylum.Actinobacteria.id.400	Late DKD in T1D	IVW	2	− 0.7768	0.31099	0.0125	0.46	0.250–0.846
phylum.Actinobacteria.id.400	Late DKD in T1D	Maximum likelihood	2	− 0.7775	0.31967	0.01501	0.46	0.246–0.860
phylum.Actinobacteria.id.400	Late DKD in T1D	cML-MA-BIC	2	− 0.7775	0.31967	0.01501	0.46	0.246–0.860
phylum.Actinobacteria.id.400	Late DKD in T2D	IVW	2	− 1.1848	0.45835	0.00974	0.306	0.125–0.751
phylum.Actinobacteria.id.400	Late DKD in T2D	Maximum likelihood	2	− 1.1890	0.47207	0.01178	0.768	0.121–0.768
phylum.Actinobacteria.id.400	Late DKD in T2D	cML-MA-BIC	2	− 1.1890	0.47207	0.01178	0.768	0.121–0.768
class.Actinobacteria.id.419	Ketoacidosis in T2D	IVW	2	2.58544	1.14887	0.02442	13.269	1.396–126.120
class.Actinobacteria.id.419	Ketoacidosis in T2D	Maximum likelihood	2	2.58704	1.16672	0.0266	13.29	1.350–130.818
class.Actinobacteria.id.419	Ketoacidosis in T2D	cML-MA-BIC	2	2.58703	1.16672	0.0266	13.29	1.350–130.817
phylum.Actinobacteria.id.400	Ketoacidosis in T2D	IVW	2	3.67128	1.45647	0.01171	39.302	2.263–682.628
phylum.Actinobacteria.id.400	Ketoacidosis in T2D	Maximum likelihood	2	3.674	1.49821	0.0142	39.409	2.091–742.841
phylum.Actinobacteria.id.400	Ketoacidosis in T2D	cML-MA-BIC	2	3.67402	1.49821	0.0142	39.41	2.091–742.858

SNP single nucleotide polymorphism, SE standard error, OR odds ratio, 95%CI 95% confidence interval, IVW Inverse-variance weighted, DKD diabetic kidney disease, T1D type1 diabetes mellitus, T2D type2 diabetes mellitus

**Table 2:** Mendelian randomisation (MR) results of causal effects between gut microbiota and diabetic complication risk (p < 1 × 10^–6^)

Gut microbiota (exposure)	Diabetic complications (outcome)	Methods	Number of SNPs	Beta	SE	p-value	OR	95% CI
order.Bifidobacteriales.id.432	DKD in T1D	IVW	5	− 0.4849	0.15813	0.00217	0.616	0.452–0.84
order.Bifidobacteriales.id.432	DKD in T1D	Weighted median	5	− 0.5795	0.19178	0.00251	0.56	0.385–0.816
order.Bifidobacteriales.id.432	DKD in T1D	Maximum likelihood	5	− 0.4892	0.16214	0.00255	0.613	0.446–0.843
family.Bifidobacteriaceae.id.433	Early DKD in T1D	IVW	5	− 0.8609	0.21931	8.7E−05	0.423	0.275–0.65
family.Bifidobacteriaceae.id.433	Early DKD in T1D	Weighted median	5	− 0.8621	0.26272	0.00103	0.422	0.252–0.707
family.Bifidobacteriaceae.id.433	Early DKD in T1D	Maximum likelihood	5	− 0.8607	0.22585	0.00014	0.423	0.272–0.658
family.Bifidobacteriaceae.id.433	Early DKD in T1D	cML-MA-BIC	5	− 0.719	0.22036	0.0011	0.487	0.316–0.75
genus.Bifidobacterium.id.436	Early DKD in T1D	IVW	5	− 0.8461	0.21513	8.4E−05	0.429	0.281–0.654
genus.Bifidobacterium.id.436	Early DKD in T1D	Maximum likelihood	5	− 0.8457	0.22142	0.00013	0.429	0.278–0.662
genus.Bifidobacterium.id.436	Early DKD in T1D	cML-MA-BIC	5	− 0.8459	0.22151	0.00013	0.429	0.278–0.662
order.Bifidobacteriales.id.432	Early DKD in T1D	IVW	5	− 0.8609	0.21931	8.7E−05	0.423	0.275–0.65
order.Bifidobacteriales.id.432	Early DKD in T1D	Weighted median	5	− 0.8621	0.26272	0.00103	0.422	0.252–0.707
order.Bifidobacteriales.id.432	Early DKD in T1D	Maximum likelihood	5	− 0.8607	0.22585	0.00014	0.423	0.272–0.658
order.Bifidobacteriales.id.432	Early DKD in T1D	cML-MA-BIC	5	− 0.719	0.22036	0.0011	0.487	0.316–0.75
phylum.Actinobacteria.id.400	Early DKD in T1D	IVW	5	− 0.8367	0.26177	0.00139	0.433	0.259–0.724
phylum.Actinobacteria.id.400	Early DKD in T1D	Weighted median	5	− 1.0301	0.32515	0.00153	0.357	0.189–0.675
phylum.Actinobacteria.id.400	Early DKD in T1D	Maximum likelihood	5	− 0.8479	0.2716	0.0018	0.428	0.252–0.729
phylum.Actinobacteria.id.400	Early DKD in T1D	cML-MA-BIC	5	− 0.8498	0.27229	0.0018	0.428	0.251–0.729
phylum.Actinobacteria.id.400	Ketoacidosis in T2D	IVW	5	3.11781	1.08287	0.00399	22.597	2.706–188.714
phylum.Actinobacteria.id.400	Ketoacidosis in T2D	Maximum likelihood	5	3.12692	1.1096	0.00483	22.804	2.591–200.686
phylum.Actinobacteria.id.400	Ketoacidosis in T2D	cML-MA-BIC	5	3.12417	1.1114	0.00494	22.741	2.575–200.84

SNP single nucleotide polymorphism, SE standard error, OR odds ratio, 95% CI 95% confidence interval, IVW Inverse-variance weighted, DKD diabetic kidney disease, T1D type1 diabetes mellitus, T2D type2 diabetes mellitus

### Reverse MR results

**Fig. 4:**
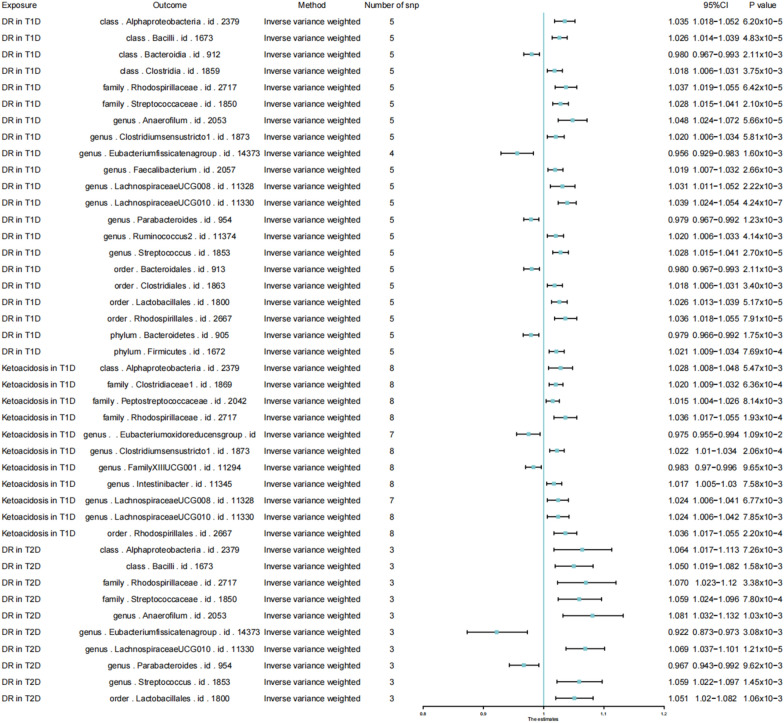
Forest plot of reverse causal effects between gut microbiota and diabetic complications (p < 5 × 10^–8^). Forrest plot representing the reverse MR estimats and 95% CI values of the causal effects of diabetic complications (as exposure) and gut microbiota (as outcomes), instrumental variables selected by p-value < 5 × 10^–8^, as estimated using Inverse-variance weighted. 95% CI 95% confidence interval, SNP single nucleotide polymorphism

**Fig. 5:**
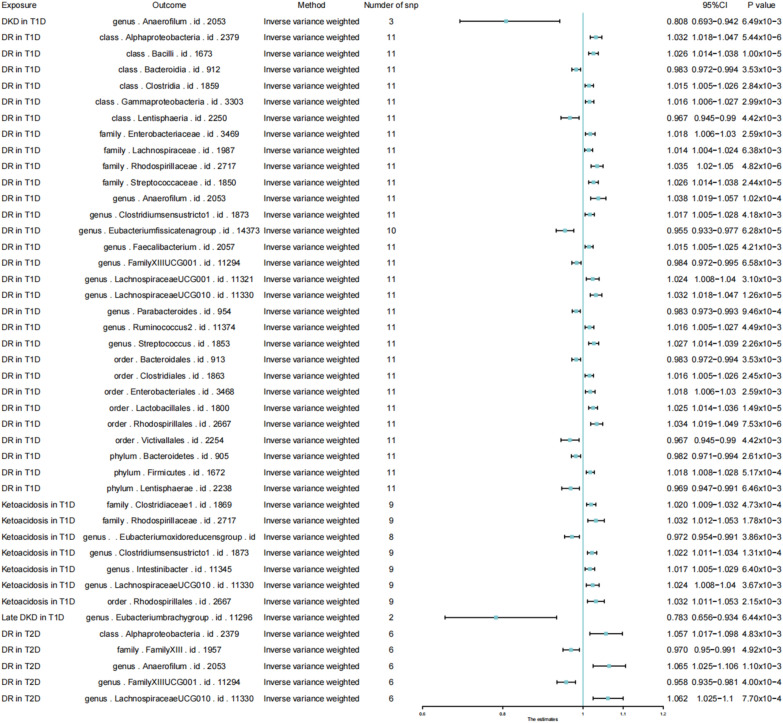
Forest plot of bi-directional MR results of the causal effects between gut microbiota and diabetic complications (p < 1 × 10^–6^). Forrest plot representing the reverse MR estimats and 95% CI values of the causal effects of diabetic complications (as exposure) and gut microbiota (as outcomes), instrumental variables selected by p-value < 1 × 10^–6^, as estimated using Inverse-variance weighted. 95% CI 95% confidence interval, SNP single nucleotide polymorphism

**Fig. 6:**
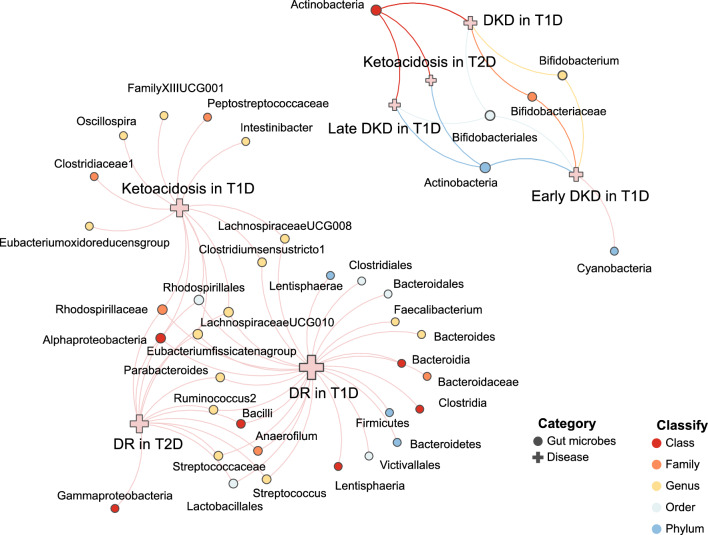
The causal relationships between gut microbiota and diabetic complications by mendelian analysis

Besides, we performed a reverse Mendelian analysis using diabetic complications as the exposure factor and gut microbiota as the outcome factor. As we can see in Fig. 4, when the threshold was set at (p < 5 × 10^–8^), mainly DR in T1D (Eubacterium fissicatena-IVW: OR = 0.956, 95% CI 0.929–0.983, p = 1.60 × 10^–3^; LachnospiraceaeUCG01-IVW: OR = 1.039, 95% CI 1.024–1.054, p = 4.24 × 10^–7^) DR in T2D(Eubacterium fissicatena-IVW: OR = 0.922, 95% CI 0.873–0.973, p = 3.08 × 10^–3^; LachnospiraceaeUCG01-IVW: OR = 1.069, 95%CI 1.037–1.101, p = 1.21 × 10^–5^), and Ketoacidosis in T1D (Eubacterium fissicatena-IVW: OR = 0.975, 95% CI 0.955–0.994, p = 0.010875207; LachnospiraceaeUCG01-IVW: OR = 1.024, 95% CI 1.006–1.041, p = 6.80 × 10^–3^) had an effect on the Eubacterium fissicatena and LachnospiraceaeUCG010's intestinal occupancy within the gut is affected. Next, in Fig. 5, we set the threshold to (p < 1 × 10^–6^) and we obtain that DR in T1D (IVW: OR = 1.024, 95% CI 1.008–1.040, p = 0.003095737), DR in T2D (IVW: OR = 1.062, 95% CI 1.025–1.100, p = 7.70E−04), and Ketoacidosis in T1D(IVW: OR = 1.024, 95% CI 1.008–1.04, p = 0.003666056) increase the percentage of LachnospiraceaeUCG010. Finally, we present the results of the bidirectional Mendelian analyses between bacteria and disease as a network diagram using the https://www.chiplot.online [21], following the results in Figs. 2 and 3. In the top right of Fig. 6, the effects of gut microbiota on disease are shown. Mainly phylum.Actinobacteria and order.Bifidobacteriale have an effect on DKD and DKA, where the cross represents the disease, the circle represents the bacteria, and the colours of the circle represent the classification of the bacteria (Phylum, Class, Order, Family, Genus). The colours of the line segments represents causality, with an adjacent graph that matches the colour of the line segment as the cause, connected to another graph that is the result. The bottom left corner shows the Mendelian result of disease on bacteria. We can see that mainly DR in T1D, DR in T2D, and Ketoacidosis in T2D are affected by three diseases on the percentage of gut microbiota. Mainly 36 bacteria are affected by the three diseases DR in T1D, DR in T2D, and Ketoacidosis in T2D.

### Sensitive analyses

There was no evidence of horizontal pleiotropy for primary and reverse MR results with p > 0.05 when using the MR-Egger regression intercept approach in two thresholds (Table S3, Table S4, Table S7, Table S8). In addition, the results of the Cochrane Q statistics showed no significant heterogeneity in primary MR for gut microbiota in diabetic complications (p > 0.05). DKA in T1D in class.Alphaproteobacteria and genus.LachnospiraceaeUCG010 (threshold of p < 5 × 10^–8^), DKA in T1D in family.Rhodospirillaceae and order.Rhodospirillales (threshold of p < 1 × 10^–6^), and DR in T2D in genus.LachnospiraceaeUCG010 (threshold of p < 1 × 10^–6^) showed heterogeneity in one of the IVW or MR Egger methods. The Maximum likelihood and cML-MA-BIC methods yielded similar causal estimates with IVW method (Table S1, Table S2, Table S5, Table S6). Thus, The MR results were also relatively reliable.

## Discussion

Gut microbiota has been richly studied in oncology, intestinal inflammation and metabolic diseases [22, 23]. However, comprehensive analyses on the association between gut microbiota and multiple diabetic complications have not emerged. In this paper, we analyzed the association between 196 gut microbiota and common diabetic complications (DKD in T1D, DKD in T2D, Early DKD in T1D, Early DKD in T2D, Late DKD in T1D, Late DKD in T2D, Ketoacidosis in T1D, Ketoacidosis in T2D, DR in T1D, DR in T2D) between bidirectional causality. Thus, we obtained two results, a causal association between gut microbiota and diabetic complications, and a causal association between diabetic complications and gut microbiota. Firstly, 2 bacterial increasing the risk of developing diabetic complications and 7 bacterial decreasing diabetic complications were obtained. Then, we found that DR in T1D, DR in T2D, and Ketoacidosis in T1D affect the intestinal occupancy ratio of 36 bacteria. These findings are instructive in reducing risk indices for diabetic complications and understanding of the relationship between gut microbiota and diabetic complications.

Actinobacteria are Gram positive, multiple branching rods, non-motile, non-spore-forming and anaerobic bacteria, that include three main anaerobe families (Bifidobacteria, Propionibacteria and Corynebacteria) and an aerobe family (Streptomyces) [24]. Actinobacteria have been shown to be associated with smoking as well as lupus erythematosus in previous Mendelian studies [25, 26]. In the Japanese study, Actinobacteria were significantly elevated in the intestinal flora of diabetic patients [27]. In our study, we found phylum.Actinobacteria to be highly associated with DKD and ketoacidosis. In DKD, Actinobacteria is a protective factor, and when the percentage of Actinobacteria is high, the complication rate of DKD is reduced. Meanwhile, order. bifidobacteriales was included in phylum. actinobacteria, also a protective factor for DKD. In an article in gestational diabetes mellitus (GDM), pregnant women in whom Bifidobacterium and Actinomycete were the predominant gut microbiota exhibited higher blood glucose levels [28]. These studies all suggest that Bifidobacterium and Actinomycete have a large potential for research in both diabetes development and diabetes complications. Perhaps, increasing the proportion of Bifidobacterium and Actinomycete in the intestinal tract of diabetic patients would be beneficial in reducing the rate of diabetes and diabetic complications.

However, we found that Actinobacteria became a highly influential (OR = 13.269 and OR = 39.302) risk factor in ketoacidosis. Diabetic ketoacidosis (DKA) is the most common acute hyperglycaemic emergency in people with hyperglycemic and occurs more often in paediatric patients with T1D [29]. Extant studies on the relationship between DKA and gut microbiota are not abundant. Some researchers have shown that a high ketogenic diet promotes Actinobacteria levels in the gut of mice, which may suggest that Actinobacteria levels are associated with ketone metabolism in vivo [30]. At the same time, it was shown that a patient with acute diabetic ketoacidosis developed Actinomyces turicensis-induced necrotising soft-tissue infection, and it was suggested that Actinomyces turicensis should be treated as the primary bacterium with topical active treatment [31]. This suggests to us that infiltration of Actinomyces turicensis flora by non-cutaneous sources penetrates the skin and induces necrotising soft-tissue infection. Indeed, many studies have demonstrated that intestinal wall permeability is enhanced in T2D, thereby increasing the likelihood of bacterial infiltration from the intestinal wall into the body [32]. Therefore, it is conceivable that the skin soft-tissue infections seen in this literature during the development of DKA could be the result of elevated Actinobacteria infiltrating from the intestines of patients with DKA and exacerbating the infection in the patient’s body. In order to prove this hypothesis, more evidence will be needed to verify whether there is a causal link between Actinobacteria and intestinal wall permeability as well as DKA. At the same time, whether the high level of phylum.Actinobacteria in DKA patients really has a direct role in the pathogenesis of DKA remains to be studied in more experimental studies. Nevertheless, the above literature, as well as the results obtained in this paper, may suggest that in clinical practice, when T2D patients are associated with high levels of phylum.Actinobacteria, one needs to be vigilant for the development of Ketoacidosis as well as in vivo infections.

In our reverse Mendelian analyses, we found that it was mainly DR in T1D, DR in T2D, and Ketoacidosis in T1D that had an effect on the occupancy of bacteria in the gut. And, it was mainly Eubacterium fissicatenaand LachnospiraceaeUCG010 that had altered occupancy. When DR in T1D, DR in T2D, or Ketoacidosis in T1D occurs, it can reduce the ration of genus. Eubacterium fissicatena; conversely DR in T1D, DR in T2D, or Ketoacidosis in T1D can increase the percentage of LachnospiraceaeUCG010. It has been shown that oral administration of Eubacterium hallii increases insulin sensitivity in diabetic mice [33]. It has also been suggested that intestinal Eubacterium can inhibit lymphoma development by decreasing TNF-a levels and thereby reducing the inflammatory response [34]. It is also suggested that increasing the percentage of Eubacterium oxidoreducens in DR in T1D, DR in T2D, or Ketoacidosis in T1D may have a protective effect against the above diseases as well as diabetes.

As diabetic retinopathy is a common complication in the later stages of diabetes and seriously affects the quality of life of patients. The number of people blinded by DR has increased from 200,000 to 400,000, and the number of people with moderate to severe visual impairment has increased from 1.4 million to 2.6 million [35–37]. In our study, two types of DRs, DR in T1D and DR in T2D, were found to be simultaneously associated with Anaerofilum, genus.Eubacterium fissicaten, Streptococcus, Parabacteroides, and Ruminococcus2, LachnospiraceaeUCG010 related. Among them, genus.Eubacterium fissicaten, Parabacteroides will be reduced in the percentage of patients with DR in T1D, DR in T2D. It has been shown that oral administration of Parabacteroides goldsteinii in High-fat diet (HFD)-fed reduces obesity, inflammation levels, and insulin resistance. At the same time, in our study, we found that DR patients were accompanied by lower levels of Parabacteroides, perhaps increasing the percentage of Parabacteroides also has a positive impact on the treatment of DR in diabetic patients. However, there exists a question as to why Parabacteroides levels are only decreased in patients with DR? In our study, the effect of Parabacteroides decline was not seen in patients with other diabetic complications, does it mean that there is a special potential therapeutic target of Parabacteroides for diabetic patients with DR? This series of questions needs to be explored in follow-up.

### Strengths and limitations

The major strengths of our study include that we utilize large-scale GWAS data to conduct MR study to exclude unknown confounders that are commonly observed in epidemiological studies, and we comprehensively analyse the bi-directional causal relationship between type 1 and type 2 diabetes mellites complications and up to 211 gut microbiotas. Nevertheless, this work also has compelling limitations. First, we explored several diabetic complications, but due to limited data access, we were unable to analyse the causal relationship between other complications and gut microbiota, such as neuropathy and cardiovascular diseases in T1D and T2D. Second, the GWAS datasets in this study were mainly from European descent, limiting the generalizability of our results globally. More research on population of non-European descent is needed. Third, because of our strict threshold (p-value < 5 × 10^–8^), the genetic liabilities of many gut microbiota was excluded at the IV selection stage, which may have led to some results being missed. To address this, we also used a threshold of p-value < 1 × 10^–6^ to verify the results. Finally, we could not completely avoid the effect of horizontal pleiotropy, despite using various sensitivity methods to mitigate it.

## Conclusions

In this MD study, we comprehensively assessed the causal relationship between ten diabetic complications and gut microbiota. MR analyses were obtained for Bifidobacterium and Actinomycete affecting DKD and DKA. Reverse MD analyses yielded that DR in T1D, DR in T2D, and Ketoacidosis in T2D affect Eubacterium fissicatena as well as LachnospiraceaeUCG010 in the gut microbiota ration. Our study suggests that there is an effect of altering the adult tract flora on diabetic complications, and that the intestinal flora also alters the flora ratio when diabetic complications occur. These results provide new perspectives for the prevention and treatment of diabetic complications. Further research is needed to confirm these findings and to understand the underlying mechanisms involved.

### Supplementary Information

Additional file 1. Sensitive analyses for the causal results of the primary Mendelian randomization. Table S1 Heterogeneity tests for the causal results of the primary Mendelian randomization (p < 5 × 10^–8^). Table S2 Heterogeneity tests for the causal results of the primary Mendelian randomization (p < 1 × 10^–6^). Table S3 Pleiotropy tests for the causal results of the primary Mendelian randomization (p <  × 10^–8^). Table S4 Pleiotropy tests for the causal results of the primary Mendelian randomization (p < 1 × 10^–6^).

Additional file 2. Sensitive analyses for the causal results of the bi-directional Mendelian randomization. Table S5 Heterogeneity tests for the causal results of the bi-directional Mendelian randomization (p < 5 × 10^–8^). Table S6 Heterogeneity tests for the causal results of the bi-directional Mendelian randomization (p < 1 × 10^–6^). Table S7 Pleiotropy tests for the causal results of the bi-directional Mendelian randomization (p < 5 × 10^–8^). Table S8 Pleiotropy tests for the causal results of the bi-directional Mendelian randomization (p < 1 × 10^–6^).

Additional file 3. F statistics of instrumental variables (IVs).

## Data Availability

No datasets were generated or analysed during the current study.
